# 

**Published:** 1890-07

**Authors:** 


### IMPORTANT NOTICE.

Hereafter all **communications** and **exchanges** intended for this Journal must be addressed and all **moneys** remitted to

THE INTERNATIONAL DENTAL JOURNAL,

716 Filbert Street,

PHILADELPHIA, PA.

